# The effects of different dietary fiber pectin structures on the gastrointestinal immune barrier: impact via gut microbiota and direct effects on immune cells

**DOI:** 10.1038/s12276-020-0449-2

**Published:** 2020-09-10

**Authors:** Martin Beukema, Marijke M. Faas, Paul de Vos

**Affiliations:** Immunoendocrinology, Division of Medical Biology, Department of Pathology and Medical Biology, University Medical Centre Groningen, Hanzeplein 1, 9713 GZ Groningen, The Netherlands

**Keywords:** Mucosal immunology, Translational immunology

## Abstract

Pectins are dietary fibers with different structural characteristics. Specific pectin structures can influence the gastrointestinal immune barrier by directly interacting with immune cells or by impacting the intestinal microbiota. The impact of pectin strongly depends on the specific structural characteristics of pectin; for example, the degree of methyl-esterification, acetylation and rhamnogalacturonan I or rhamnogalacturonan II neutral side chains. Here, we review the interactions of specific pectin structures with the gastrointestinal immune barrier. The effects of pectin include strengthening the mucus layer, enhancing epithelial integrity, and activating or inhibiting dendritic cell and macrophage responses. The direct interaction of pectins with the gastrointestinal immune barrier may be governed through pattern recognition receptors, such as Toll-like receptors 2 and 4 or Galectin-3. In addition, specific pectins can stimulate the diversity and abundance of beneficial microbial communities. Furthermore, the gastrointestinal immune barrier may be enhanced by short-chain fatty acids. Moreover, pectins can enhance the intestinal immune barrier by favoring the adhesion of commensal bacteria and inhibiting the adhesion of pathogens to epithelial cells. Current data illustrate that pectin may be a powerful dietary fiber to manage and prevent several inflammatory conditions, but additional human studies with pectin molecules with well-defined structures are urgently needed.

## Gastrointestinal immunity: Pectin protection

The dietary fiber pectin plays several immunity-related roles in the gastrointestinal tract and could help manage inflammatory conditions according to a review of recent research. Pectin, found in many fruits and vegetables, takes various structural forms that have different impacts on gut health. Martin Beukema and co-workers at University Medical Centre Groningen in The Netherlands reviewed how these pectin structures interact with the gastrointestinal immune barrier. Studies have shown that pectins can strengthen mucous membranes and epithelial tissue, and activate or inhibit immune cells such as macrophages. Pectins also have a positive influence on the diversity and abundance of microbiota that inhabit the gut, for example helping beneficial bacteria to adhere to epithelial cells while blocking the adhesion of pathogens. Beukema and co-workers point to an urgent need for more studies in humans.

## Introduction

Dietary fibers play an important role in the maintenance of health^1^. Several meta-analyses have illustrated that higher dietary fiber intake is correlated with a reduced incidence of disease and mortality^1–3^. These findings are corroborated by other studies showing that reduced dietary fiber intake is correlated with a higher incidence of Western diseases with anomalies in immunity^4,5^, indicating that dietary fiber plays an important role in immune homeostasis. One mechanism by which fibers may protect against the development of disease is by preserving the gastrointestinal immune barrier. The gastrointestinal immune barrier is the gatekeeper of the human body. It is composed of a mucus layer and a layer of epithelial cells and prevents luminal molecules from entering the underlying lamina propria containing immune cells^6,7^. Intestinal barrier dysfunction may result in immune disorders such as autoimmune and inflammatory diseases, including Crohn’s disease and ulcerative colitis^8,9^. Improving intestinal barrier function by increasing dietary fiber consumption may therefore be an effective strategy to prevent or delay Western immune-related diseases^10^.

Before dietary fibers are degraded by microbial enzymes in the colon, they may interact directly with the cells of the immune barrier in the small intestine^11^. The small intestine contains a thin and loose mucus layer^12^ that not only facilitates nutrient uptake but also allows dietary molecules such as fibers to interact with the intestinal epithelial and immune cells directly^11^. The consequences of these direct interactions with cells of the intestinal immune barrier include strengthening of the mucus layer^13^, enhancing barrier function of epithelial cells^14^ or modulating intestinal immune responses^15^. This direct interaction between dietary fibers and the intestinal immune system may be one of the mechanisms by which dietary fibers improve health and prevent disease^16^.

**Fig. 1:**
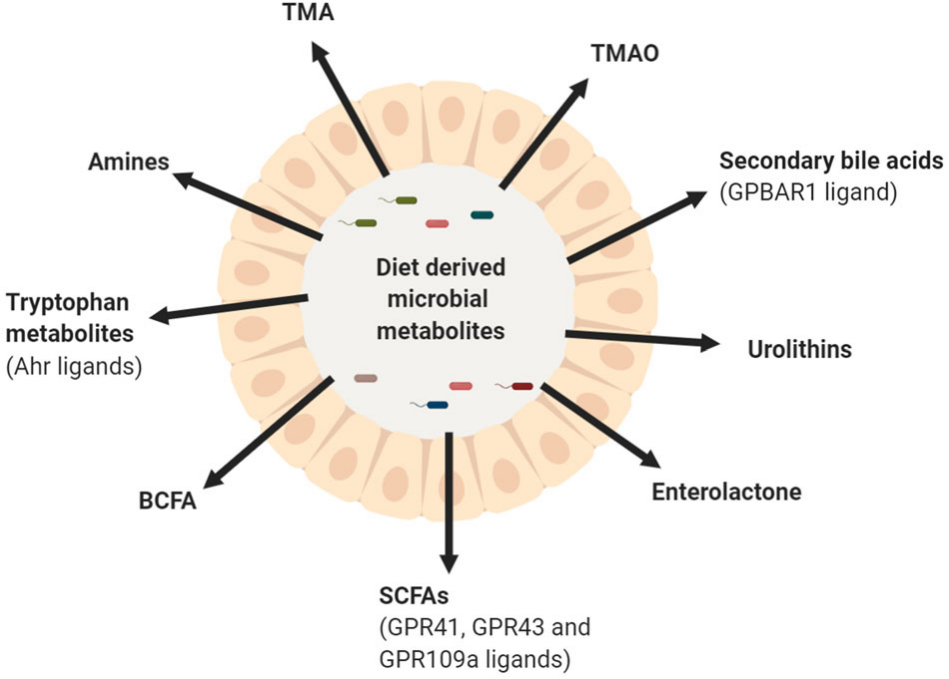
Diet-derived microbial metabolites. Intestinal microbiota can secrete diet-derived metabolites, which can potentially activate the immune system through Ahr, GPRs or GPBAR1. Ahr Aryl hydrogen receptor, BCFA branched chain fatty acid, GPBAR1 G protein–coupled bile acid receptor 1, GPR G-protein-coupled receptor, SCFA short-chain fatty acids, TMA trimethylamine, TMAO trimethylamine N-oxide.

Dietary fibers can also have indirect beneficial effects on the gastrointestinal immune barrier through stimulation of the growth of intestinal microbiota communities and their metabolic activities^17^. Dietary fibers cannot be digested by human enzymes and are subject to fermentation by the intestinal microbiota mainly in the colon^18^. Some dietary fibers stimulate the growth of specific microbial communities and serve as substrates for the production of fermentation products, such as short-chain fatty acids (SCFAs)^17^. Dietary fibers can exert health-promoting effects through stimulation of the production of SCFAs. Numerous recent publications have shown that these SCFAs play an important role in metabolism^19,20^ and immunity^21,22^. In addition to the fermentation products of dietary fibers, microbial communities can secrete other metabolites, such as tryptophan metabolites or secondary bile acids, which have the potential to regulate intestinal immunity (Fig. 1)^23^. In addition, stimulating the growth of commensal microbiota by dietary fibers may be beneficial for limiting the growth of pathogens that adhere to the intestinal wall less in the presence of a high abundance of gut commensals^24^. Dietary fibers are therefore also proposed as a means to indirectly protect the gastrointestinal immune barrier from damage caused by pathogens^24^.

The soluble dietary fiber pectin is an important dietary fiber with known modulatory effects on the gastrointestinal immune barrier. Pectins have been isolated from the primary and secondary cell walls of many fruits and vegetables, including citrus fruits, apples, sugar beets, and potatoes^25^. They mainly consist of linear 1,4-D-galacturonan (homogalacturonan) segments and branched rhamnogalacturonan segments^25^. Pectins can have many different structural characteristics contributing to their functional properties, including the degree of methyl-esterification, molecular weight or neutral side chain structures^25^. Several studies involving animal models and human subjects have shown that pectins protect the intestinal barrier from damage^26–28^. These effects may be exerted by direct interactions with the gastrointestinal immune barrier, as some types of pectins are known to interact with immune receptors^29,30^. Furthermore, pectins may protect the intestinal barrier indirectly by stimulating the growth and diversity of microbiota communities as pectins are known to stimulate microbial communities^31^. These effects are strongly dependent on the chemical structure of pectins^29–31^. In recent years, many studies have demonstrated the impact of specific structural features of pectins on their health benefits to consumers^32^. The main aim of this review article was to overview the current knowledge of the modulatory properties of specific chemical structures of pectins and their beneficial effects on the gastrointestinal immune barrier; for example, for a dietary fiber that might be instrumental in preventing many Western diseases^5^. We will focus on pectin structures that influence the immune system by direct effects on immune cells or by indirectly interacting with the immune system by modulating intestinal microbial communities and the metabolites they produce. This article may provide insight into the structural features responsible for intestinal health benefits. Ultimately, this might lead to tailoring of pectin formulation or products that might contribute to prevention or delay of human disease development.

## Differences in the chemical structures of pectins

**Fig. 2:**
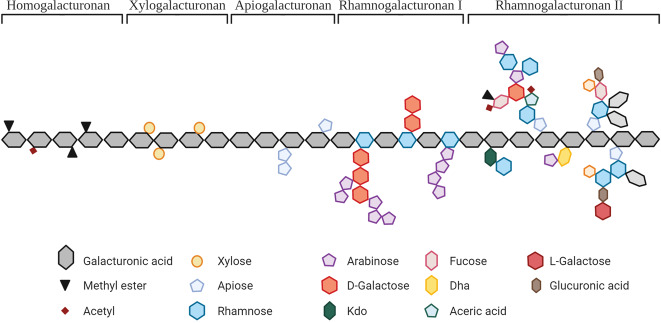
Structural characteristics of pectin molecules. A pectin molecule can contain different structural characteristics, including homogalacturonan, xylogalacturonan, apiogalacturonan, rhamnogalacturonan I, and rhamnogalacturonan II.

Pectins have been isolated from the primary and secondary cell walls of many fruits and vegetables. The structure of pectin is dependent on several parameters, including the origin, the endogenous enzyme activity or the method of extraction^25^. Pectins are heteropolysaccharides consisting mainly of α-1,4-linked galacturonic acid (GalA) residues (Fig. 2). Some pectins consist of regions with only GalA molecules, which are known as homogalacturonans. Up to 70% of pectin molecules consist of homogalacturonan regions. In those regions, the GalA residues can be methyl-esterified at the C6 carboxyl group. GalA residues in pectins can also be acetylated at O-2 or O-3, as is commonly found in sugar beet pectins^25,33^. A certain percentage of the GalA residues of pectins can have attached methyl esters, and this percentage is known as the degree of methyl-esterification (DM). Pectins can be classified as low DM pectins (DM < 50%) or high DM pectins (DM > 50%)^34^. These methyl-esterified GalA residues can be differently distributed along the pectin molecule. The degree of blockiness (DB) determines the distribution of non-esterified galacturonic acid residues. Pectins with a high DB contain a more blockwise distribution of non-esterified GalA residues, whereas low DB pectins have a more random distribution of non-esterified GalA residues^35^. Homogalacturonan pectins have been isolated from several fruits and vegetables origins, including sunflower, citrus fruits, sisal, rice endosperm cell walls or apples^33^.

In addition to homogalacturonan regions, pectins can contain other structural regions, such as xylogalacturonan, apiogalacturonan, rhamnogalacturonan I (RG-I) or rhamnogalacturonan II (RG-II) (Fig. 2). Xylogalacturonan regions are homogalacturonan structures substituted with xylose molecules. Apiogalacturonan regions are homogalacturonan structures with attached monosaccharide or disaccharide apioduranosyl. RG-I regions are based on disaccharide backbone structures of alternating galacturonic acid and rhamnose residues. The rhamnose residues in RG-I can be branched with neutral side chains consisting of galactose or arabinose. The GalA residues of RG-I are mostly non-methyl esterified. RG-II regions contain a different backbone than RG-I regions and consist of only galacturonic acid residues. RG-II molecules have complex pectin structures that may comprise up to 12 different branched sugar residues. These sugar molecules can be linked by 21 different glycosidic linkages, making RG-II molecules very complex^25,36^. Pectins containing regions with such a high number of neutral side chains have been isolated from many fruits and vegetables, including apples, sugar beets, apricots, carrots, cabbage, onions or pears^33^.

## The gastrointestinal immune barrier as a primary target for pectins

**Fig. 3:**
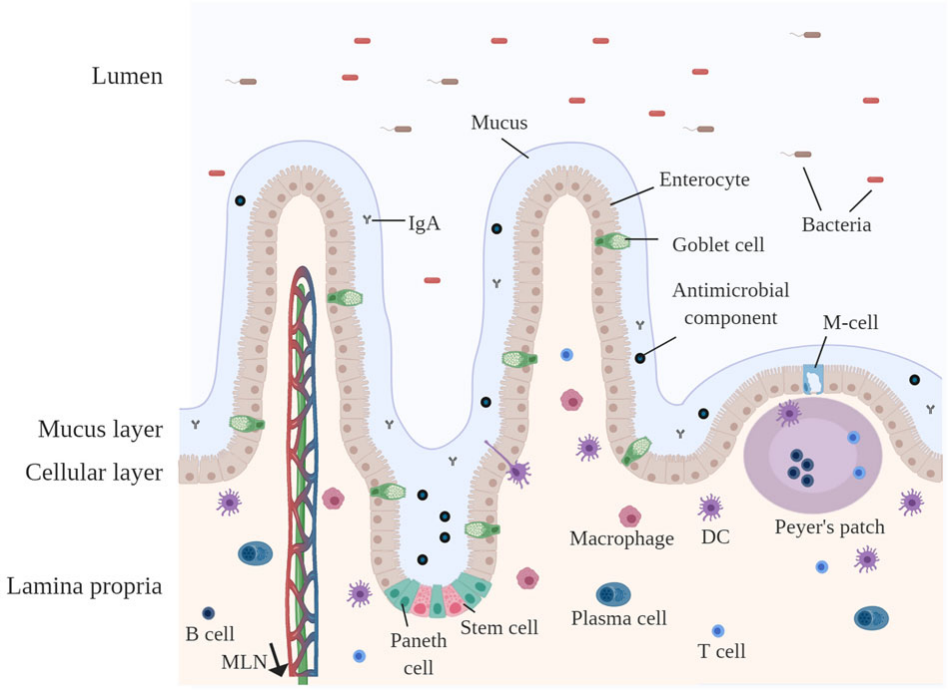
The gastrointestinal immune barrier. The gastrointestinal immune barrier contains several layers that provide protection against harmful substances. From the outside (lumen) to the inside (lamina propria): (1) The mucus layer in the small intestine consists of a single loose layer, whereas in the colon, it consists of a loose outer layer and a dense inner layer. The mucus layer provides extra protection against pathogens due to the presence of IgA and antimicrobial peptides. (2) The cellular layer consists of a single layer of epithelial cells that forms a physical barrier against incoming antigens. (3) Beyond the cellular layer is the lamina propria, which is densely populated with immune cells. These immune cells provide protection against harmful substances but also induce tolerance against harmless substances.

Pectins are considered to greatly impact the health of the gastrointestinal tract^37^, although some studies have reported effects independent of those exerted on the gastrointestinal tract, including the direct uptake of pectic oligosaccharides in blood and the direct interaction of pectins with systemic organs^38^. Currently, however, the pronounced health benefits of pectins are considered to result from modulating the intestinal immune barrier^37^. As pectins might impact different aspects of the gastrointestinal immune barrier^37^, we first briefly discuss the current understanding of the composition of the gastrointestinal immune barrier. The gastrointestinal immune barrier consists of multiple layers, including a mucus layer, a cellular layer of epithelial cells and the lamina propria containing immune cells (Fig. 3).

The mucus layer forms a physical barrier that separates the epithelium from the microbiota and luminal content^39^. In addition, it provides a substrate for microbial growth^24,40^. These bacteria secrete fermentation products that alter the composition and gelling properties of the mucus layer^40^. These mucus strengthening effects are also induced by luminal dietary components, including pectins^41^. The mucus layer is composed of secretory products from goblet cells, Paneth cells and other epithelial cells^42^. The main components of the mucus layer are mucins, which are goblet cell-derived glycoproteins^42^. Luminal bacteria or dietary components can stimulate the secretion of mucins from goblet cells by activating pattern recognition receptors (PRRs) or stimulating SCFA production^43^. Mucin secretion from goblet cells can also be enhanced by immune cell-derived cytokines^44^. In addition to mucins, the mucus layer contains immunoglobin A (IgA) and Paneth cell-derived antimicrobial components, which provide a defense against invading, undesired microbial species^45^. Luminal bacteria or dietary components can stimulate the secretion of these antimicrobial components after PRR activation in Paneth cells^46^.

Underneath the mucus layer resides the cellular layer, which is composed of a single layer of epithelial cells and intraepithelial lymphocytes. The intraepithelial lymphocytes secrete factors that support and maintain the epithelial layer^47^. The epithelial cells consist of enterocytes and secretory epithelial cells, such as the aforementioned Paneth cells or goblet cells^48^. Furthermore, the cellular layer contains an additional epithelial cell type called microfold (M) cells, which are present on subepithelial lymphoid structures called Peyer’s patches (PP). M cells are specialized antigen sampling cells that present antigens to underlying immune cells in the PP^49^. Together, these epithelial cells form a physical barrier that separates the luminal content from the lamina propria. The integrity of the gastrointestinal barrier relies on the permeability of the epithelial cell layer^50^. A network of desmosomes, tight junctions and adherens junctions regulates epithelial permeability and thereby the paracellular passage of the luminal content to the underlying lamina propria^50^. Epithelial integrity can be enhanced by bacteria or luminal dietary components through the activation of PRRs, such as TLR2, or through SCFA-induced GPR activation^51,52^.

A large proportion, 70–80%, of all immune cells in the human body are located in gut-associated lymphoid tissues (GALT), which include the lamina propria, PP and mesenteric lymph nodes (mLN)^39^. These immune cells must recognize incoming antigens and provide protection against harmful substances, but also induce tolerance for commensal bacteria^53^. The lamina propria is the homing site for a large proportion of innate immune cells, such as DCs, macrophages, innate lymphoid cells and granulocytes, and adaptive immune cells, such as B cells and T cells^39^. DCs and macrophages are innate immune cells that can recognize luminal antigens with PRRs^54^. DCs are capable of sampling antigens and food components through their epithelial-penetrating dendrites^55^. After recognition, DCs and macrophages can phagocytose microbes or other molecules and present them as antigens to adaptive immune cells in the lamina propria, PP or mLN. Via this route, DCs and macrophages regulate the induction of IgA-producing plasma cells or induce the differentiation of naïve T cells into regulatory T cells (Treg) or effector T cells (Th1, Th2, Th9, Th17, or Th22)^56^. In addition, the differentiation of naïve T cells can be induced by microbial-derived SCFAs or aryl hydrogen receptor ligands^57^. These effector T cells induce pro-inflammatory responses against specific antigens, whereas their counterparts, Tregs, suppress these pro-inflammatory responses^57^.

PPs are located in the small intestine and are located under the epithelium. PPs consist of DCs, macrophages, B cells, and T cells. Luminal antigens from the microbiota or dietary components can be taken up through M cells and are presented to subepithelial dendritic cells^49^. DCs present luminal antigens to underlying adaptive immune cells, leading to specific T cell differentiation or the formation of IgA-producing plasma cells^58^.

The immune cells in the lamina propria and in the PP can communicate with immune cells in the mLN. CD103^+^-expressing DCs mainly migrate to the mLN via lymph vessels, after which they stimulate naïve T cells and B cells. CD103^+^-expressing DCs together with mLN stromal cells generate gut-homing T cells^59,60^.

A balanced regulation between pro-inflammatory and anti-inflammatory responses in the gastrointestinal tract leads to protection against pathogens and tolerance to food antigens or commensal microbiota^61^. However, tolerance to dietary antigens appears to develop in a different compartment of the gastrointestinal tract than tolerance to intestinal microbiota^61,62^. This is because intestinal immunity in the small intestine is more directed towards exposure to dietary antigens, whereas immune responses in the large intestine are more adapted to the greater bacterial load^61,62^. Pectins may therefore stimulate the immune system in the small intestine through direct interactions with immune cells, whereas they may stimulate large intestinal immunity in a microbiota-dependent manner.

## Interaction of pectins with immune cells and their receptors

Dietary fibers such as pectins can impact the aforementioned gastrointestinal immune barrier at different levels, as discussed in the following sections.

### Strengthening of the mucus layer

Dietary fibers such as pectins can strengthen the mucus layer through several mechanisms^63^. A strengthened mucus layer may limit the passage of harmful substances into the underlying tissues and thereby prevent activation of inflammatory responses. Several studies have shown that pectins have beneficial effects on the integrity of the gastrointestinal mucus layer. Under both healthy and diseased conditions, it has been shown that pectins can strengthen the intestinal mucus layer by influencing goblet cells or by the mucoadhesive effects of pectins^41,64–66^.

The effects of pectins on the mucus layer are dependent on the structure of pectin^41,64,65^. In vitro, both DM30 and DM59 pectins stimulated mucin secretion in HT-29MTX cells^41^. However, in vivo in rats, only DM30 pectins, and not DM59 pectins, were able to stimulate jejunal mucus secretion by goblet cells without affecting the number of mucus-secreting goblet cells^41^. The different effects of low and high DM pectins on mucus secretion could be explained by the different mucoadhesive properties of low and high DM pectins. Due to the negative charge of their non-esterified galacturonic acid residues, DM38 pectins cannot interact with the negatively charged mucins^65^. Low DM pectins are thereby able to penetrate the mucus layer and reach the epithelium, where they may stimulate the production and secretion of mucus by goblet cells^67^. DM70 pectins, however, can form hydrogen bonds with mucins and form gel networks that strengthen the mucus layer^65^. High DM pectins are not thought to reach goblet cells to induce mucus secretion, but they may protect the epithelium from harmful mucus-penetrating agents by strengthening the mucus layer. In addition, the pectin structure of RG-I showed protective effects on the mucus layer in mice with DSS-induced colitis^66^. RG-I protected the mucus layer by preserving the number of goblet cells and the expression of Muc-1^66^. Together, these findings suggest that low DM pectins, high DM pectins or RG-I pectin structures can strengthen gut barrier function by stimulating mucin secretion by goblet cells or through mucus adhesive properties.

### Preservation of epithelial integrity

Dietary fibers are known to have positive effects on epithelial cell layer integrity in the gastrointestinal tract. They can enhance epithelial integrity by stimulating gut bacteria to produce SCFAs that are derived from microbial fermentation of dietary fibers or through direct interaction with pattern recognition receptors (PRRs), such as TLR2^14,68^. Several studies have highlighted the importance of pectins in the preservation of epithelial integrity under healthy and diseased conditions^27,28,69^. In healthy young adults and elderly individuals, four weeks of pectin supplementation did not increase epithelial barrier integrity, as tight junction-related genes were unchanged after pectin administration^28^. Under diseased conditions, pectins lowered epithelial permeability in Bangladeshi children with persistent diarrhea^69^. In addition, pectins were able to maintain epithelial integrity in rats after disruption by a high-fat diet^27^. These studies suggest that pectins cannot enhance epithelial integrity under healthy conditions, but they can restore or maintain epithelial integrity under diseased conditions.

Recent research indicates that not all pectins but specific structural characteristics of pectins induce the protective effects of pectins on epithelial integrity, which might cause sometimes contradictory findings in humans. DM63 pectins were not able to enhance epithelial integrity in intestinal biopsies from healthy young adults and elderly individuals using Ussing chambers^28^. However, Vogt et al. demonstrated in vitro that DM30 or DM74 pectins were more protective against the epithelial barrier disruptor PMA than DM56 pectins^29^. Moreover, DM7 pectins maintained epithelial integrity in mice with caerulein-induced pancreatitis by preserving tight junction structures^26^. The findings of Vogt et al. and Sun et al. suggest that compared to DM63 pectins, low DM pectins (DM < 30) or very high DM pectins (DM > 74) might enhance epithelial integrity in young adults and elderly individuals^28^. In addition to the DM-dependent effects, the in vitro effect of RG-I pectins was investigated. RG-I pectins reduced intestinal permeability in Caco-2 cells by maintaining claudin-1 structures^66^. These studies demonstrate that pectins can protect epithelial integrity against barrier-disrupting agents by maintaining tight junction structures. Low DM pectins and very high DM pectins or pectins with RG-I structures may be responsible for these effects^26,29,66^.

### Pectin and innate immune responses after uptake in Peyer’s patches

Many dietary fibers, including pectins, can directly interact with innate immune cells, such as macrophages or DCs, and influence their responses^70–73^. It has been suggested that dietary fibers are transported into the PP through M cells and activate immune cells, and this effect has been demonstrated for pectins^70,74–76^. In addition, in vitro studies have demonstrated that pectins activate or inhibit the responses of DCs and macrophages through direct interaction with immune receptors^71–73,77^. The activation and inhibition of macrophage and DC responses may be beneficial to the host under different circumstances. Under healthy conditions, mild activation of innate immune responses by pectins can be beneficial because it promotes intestinal immunity. Furthermore, under conditions with aberrant immune activation, inhibition of immune activation can be beneficial^78^. Attenuating immune activation may prevent the initiation of excessive immune responses and thereby limit inappropriate activation of immune cells and inflammation^78^.

Several in vitro studies have demonstrated that specific pectin structures can activate DC and macrophage responses^73,76,77^. Suh et al. demonstrated that pectins must have an intact backbone to exhibit their activating effects, as pectin hydrolysates with low molecular weights have no activating properties^76^. Furthermore, other studies demonstrated that RG-I and RG-II pectins activate macrophages and dendritic cells, but RG-I pectins activate these cells more than RG-II pectins^73,77^. Galactan and arabinan structures seem to regulate the strong activation of immune responses by RG-I pectins^73,77^. It has also been demonstrated that the DM of pectin plays an important role in immune activation. Partial removal of methyl esters from DM57 pectins to DM21 pectins enhanced the macrophage-activating properties of pectins^72^. This was, however, not corroborated by another study that showed that partial removal of methyl esters from DM85 pectins to DM17 pectins strongly decreased its macrophage-activating effects^79^. This could, however, be explained by activating properties of RG-I side chains in this DM85 pectin, which were removed together with the methyl esters by a specific procedure^79^. Current insight is that the molecular weight, DM and RG-I side chain structures of pectin influence the ability of pectins to activate macrophages and DCs. RG-II side chains may be involved but activate immune cells to a lower extent^72,73,76,77,79^.

Several in vitro studies have demonstrated that specific pectin structures can inhibit DC and macrophage responses^70–72^. Ishisono et al. suggested that the highly branched side chains of pectins are required for the anti-inflammatory effects of pectins on LPS-induced IL-6 secretion in macrophages^70^. This study, however, did not specify which specific side chains of RG-I or RG-II pectins were required. Another study showed that removing the RG-I side chains of pectins enhanced the inhibitory effect of pectins on LPS-induced cytokine secretion in macrophages^71^. The anti-inflammatory effects as measured by Ishisono et al. may therefore have been derived from RG-II side chain structures. Furthermore, another study confirmed that DM21 pectins, with a certain degree of acetylation of 9%, have anti-inflammatory properties and inhibit macrophage responses^71^. Together, these studies suggest that the inhibitory effect of pectins is orchestrated through structural characteristics such as DM, acetylation, RG-I, and RG-II structures.

### Interaction of pectin with pattern recognition receptors

Innate immune cells can recognize dietary fibers through interaction with extracellular PRRs. PRRs function as sensors that recognize molecular patterns from microbes, known as pathogen-associated molecular patterns (PAMPs). PRRs are expressed by innate immune cells, such as macrophages, dendritic cells, neutrophils and epithelial cells^80^. Molecules derived from damaged cells are known as damage-associated molecular patterns (DAMPs). PRRs play an important role in initiating innate immune responses^80^. In recent years, many studies have reported that PRRs interact with dietary fibers^80–82^. Pectins are known to affect immune responses through interactions with the PRRs galectins and Toll-like receptors^29,83^.

Pectin has been reported to bind to Galectin-3^83^. Galectin-3 is expressed extracellularly or intracellularly by different cell types and plays a role in health and disease^84^. Extracellular Galectin-3 is known to interact with carbohydrates, but intracellular Galectin-3 is considered to bind to intracellular proteins^84^. The effects of pectins on Galectin-3 are suggested to initially occur via extracellular Galectin-3. However, as pectin and Galectin-3 can be endocytosed by macrophages, pectins may also affect intracellular pathways by binding to Galectin-3^85^. The binding of pectins to Galectin-3 is established through the interaction between the lectin domain of Galectin-3 and galactose residues or arabinan side chains of RG-I and RG-II pectins^83^. By binding to Galectin-3, pectins can stimulate immunity against cancer cells. Cancer cells express Galectin-3, limiting T-cell mediated immunity against cancer cells. Galectin-3 binds to T-cells, leading to inhibition of T cell responses or apoptosis. By binding to Galectin-3, pectins block the inhibition of T-cell mediated immunity against cancer cells and can therefore be used in anticancer therapy^86,87^. In addition to cancer cells, Galectin-3 is expressed on immune cells, such as monocytes, macrophages, DCs, neutrophils and epithelial cells. These immune cells use Galectin-3 as a PRR to induce innate immune responses against pathogens^88^. Pectins might enhance these responses by binding to Galectin-3. Thus, RG-I and RG-II pectins with galactose or arabinan structures can bind to Galectin-3, and pectins might exert anti-metastatic or anti-pathogenic effects through this interaction^86–88^.

Toll-like receptors are another type of PRR that can interact with pectins. Toll-like receptors are intracellularly or extracellularly expressed on most immune and epithelial cells^51,89^. Specific pectin structures can activate signaling of TLR2 and TLR4, which are extracellularly expressed, and thereby stimulate innate immune responses. Pectins containing RG-II and homogalacturonan structures induce strong activation of TLR4 in DCs^90,91^. The TLR4-activating effect of homogalacturonan pectins is independent of the DM, as DM30, DM56 and DM74 pectins activate TLR4 at a similar level^29^. In contrast to TLR4, TLR2 activation by pectins is exerted in a DM-dependent manner. Only very high-DM pectins (DM74) activate TLR2, whereas lower DM pectins (DM30 and DM56) do not activate TLR2^29^. This indicates that the activation of TLR2 is induced after recognition of very high-DM pectins. TLR4 may be induced after recognition of the specific patterns of the non-esterified galacturonic acid backbone or RG-II structures.

In addition to their TLR-activating properties, pectins are recognized for their TLR-inhibiting properties on TLR2 and TLR4. Several studies have shown that pectins inhibit LPS-induced TLR4 activation in monocytes or dendritic cells^70,92^. These TLR4-inhibiting effects were suggested to be exerted through neutral side chains of RG-I or RG-II structures^70^. In addition, the DM can explain the TLR4-inhibiting effects of pectins because very high DM (DM90) pectin inhibited LPS-induced TLR4 activation in macrophages more effectively than lower DM pectins (DM60 and DM30)^93^. Furthermore, an opposite trend was observed for TLR2-1 inhibition. TLR2-1 was more effectively inhibited by low DM pectins, and this TLR2-1-inhibiting effect decreased with higher DM pectins^30^. Together, these results suggest that TLR4 inhibition is exerted through recognition of high DM structures or neutral side chain structures of RG-I or RG-II pectins. TLR2-1 is more effectively inhibited by low DM pectins^30^.

## Impact of pectins on gut microbiota and microbial metabolic products

The gastrointestinal tract harbors trillions of microorganisms, including bacteria, fungi, viruses, protozoa and archaea, which together form the intestinal microbiota^18^. These intestinal species interact with the intestinal immune barrier, contributing to the health status of the host^18^. Given the importance of health, maintaining a balanced intestinal microbiota composition is crucial. Dysbiosis of intestinal microbiota composition may disturb host-microbe interactions and contribute to the development of diseases^5^. A balanced composition of the intestinal microbiota is therefore vital to maintain health and prevent disease development.

Dietary fibers, including pectin, are components that can contribute to a balanced microbiota composition. Dietary fibers can have beneficial effects on the intestinal immune barrier via the intestinal microbiota through several mechanisms^94^. For example, dietary fibers can increase the abundance of beneficial intestinal microbiota^17^. As a consequence, dietary fibers reduce the abundance of pathogens or mucin-degrading bacteria, which in turn protect the epithelium against invading pathogens, as has been shown for *Citrobacter rodentium* in a mouse study^24^. Another mechanism by which dietary fibers have beneficial effects on the intestinal immune barrier is through stimulating gut bacteria to produce SCFAs^18^. Dietary fibers can be used by anaerobic bacteria as fermentation substrates to produce SCFAs, including acetate, propionate and butyrate^18^. SCFAs help maintain a low pH in the intestine, thereby inhibiting pathogen growth and favoring the growth of healthy intestinal microbiota^95^. In addition, SCFAs can dampen immune responses by acting on DCs, macrophages, neutrophils, T regulatory cells and epithelial cells^21^. They influence immune responses through interactions with GPR41, GPR43 or GPR109a, by inhibition of histone deacetylases or by direct interaction with transcription factors^96,97^. The influence of SCFAs on epithelial cells is mainly through stimulation of butyrate production in bacteria, as epithelial cells use butyrate as an energy substrate that stimulates epithelial cell growth^98^. Moreover, dietary fibers can have beneficial effects on the intestinal immune barrier, favoring adhesion of commensal or beneficial bacteria to the epithelium^99,100^. Thus, dietary fibers can have beneficial effects on the intestinal immune barrier through stimulation of microbial communities and SCFA production and modulation of bacterial adhesion to epithelial cells^18,24,99,100^. Proof that pectin can influence gut microbiota via one or a combination of the above described mechanisms is discussed in the next sections.

### Stimulation of microbiota communities by pectins

Many studies have demonstrated that dietary pectin can influence the composition and diversity of the gut microbiota^27,101–107^. The genera *Bacteroides* and *Prevotella* are the primary pectin degraders, as they possess carbohydrate-active enzymes (CAZymes) within their polysaccharide utilization loci (PUL)^108^. These bacteria use lyases, methylesterases and acetylases to breakdown pectin molecules^109^. The breakdown substrates can be used by surrounding bacteria as growth substrates and thereby influence the composition of the microbial community^110^. Several in vitro and in vivo studies have reported that pectins induce distinct microbial compositions^27,101–107^. In vitro, it was shown that pectins can increase the abundance of *Lactobacilli*, *Bacteroides* and *Prevotella*^101–103^. Furthermore, some in vivo studies showed that pectins increase the abundance of Bacteroidetes, Clostridiales, *Bacteroides*, *Lactobacilli* and *Prevotella*, whereas other in vivo studies report that pectins decrease the abundance of *Lactobacillus* and *Bacteroides* spp.^27,104–107^. The inconsistencies in the composition of the microbial communities between the different studies may be related to the variations in initial microbiota composition, fermentation substrates or the influence of the host; however, a recent correlation study found that the impact by which pectins affect the composition of the microbial community strongly depends on the structural characteristics of pectins^31^.

In a recent in vitro fermentation study, five structural characteristics of pectins were strongly linked to shifts in microbiota composition. The five structural characteristics included the DM of homogalacturonan regions, the composition of neutral sugars, the distribution of homogalacturonan and rhamnogalacturonan fractions, the degree of branching and the presence of amide groups^31^. This study suggested that DM is the most important regulator of microbiota composition^31^. The effect of DM has been demonstrated in other in vitro and in vivo studies^107,111,112^. DM8 and DM35 pectins were preferably metabolized by gut microbiota and induced a higher abundance of *Bifidobacteria* and *Bacteroides* than DM66, DM71 and DM93 pectins in fermentation studies^111,112^. In addition, both DM29 and DM53 pectins induced a higher abundance of *Prevotella* spp. and a lower abundance of *Lactobacillus* in the pig colon, but these effects were stronger with low DM pectins than with high DM pectins^107^. The differences in microbiota composition between low and high DM pectins may be related to their differences in digestibility. Low DM pectins are digested faster than high DM pectins, which is suggested to be due to the production of pectate lyases and methyl esterases that digest low DM pectins faster than high DM pectins^112^. Enzymatic degradation higher in the gastrointestinal tract of low DM pectins may lead to earlier availability of growth substrates for the microbiota than high DM pectins. Consequently, low DM pectins start inducing the growth of microbiota communities earlier in the gastrointestinal tract than high DM pectins^112,113^. In addition to DM-dependent effects of pectins, other in vitro fermentation studies confirmed that the RG-I structures and RG-I side chain structures, i.e., arabinan, galactan, oligoarabinosides, and oligogalactosides, of pectins were very efficient in stimulating the growth of *Bifidobacteria*^101,113^. Together, these results show that pectins can induce shifts in microbiota composition and that these shifts are mainly dependent on the DM or RG-I structures of pectins.

### Stimulation of SCFA production by pectins

SCFAs are metabolic products derived from the fermentation of dietary fibers under anaerobic conditions^95^. Particularly, Bacteroidetes and Firmicutes can utilize dietary fibers and are the main producers of the SCFAs acetate, butyrate and propionate in the gastrointestinal tract^95,114,115^. The generation of SCFAs strongly depends on substrate availability, microbiota composition and intestinal transit time^95^.

Pectins can be used as fermentation substrates to stimulate the production of SCFAs^31,112,113,116,117^. Each individual structural pattern of pectin induces distinct profiles of acetate, propionate and butyrate in the gastrointestinal tract. Larsen et al. showed that after in vitro fermentation of RG-I structures, high levels of propionate and butyrate were induced^31^. This effect of RG-I structures on SCFA production was confirmed in rats, showing an increased the production of SCFAs production in rats fed with RG-I-enriched diets^113^. Furthermore, a correlation study by Larsen et al. confirmed an association between high DM pectins and high levels of propionate^31^. The higher production of propionate induced by DM53 pectins compared to DM29 pectins was also reported in another fecal batch fermentation study^116^. In contrast, an opposite pattern was observed in rats, showing that total SCFA concentrations were higher in the cecum of rats fed with low DM pectins (DM0, DM1, DM35, DM36, and DM40) than in rats fed with high DM pectins (DM56, DM58, DM63, DM64, DM69, DM71, and DM93)^112,117^. The contradicting findings on the impact of pectin structures on SCFA production may result from a different initial microbiota composition, a different pectin dosage, a difference in pectin digestibility or the influence of the host on microbiota composition^31^. Overall, the current understanding is that pectins can be used as fermentation substrates for gut microbiota and that the production of SCFAs is strongly influenced by the RG-I structures or the DM of pectins.

### Effects on bacterial adhesion to epithelial cells

Dietary fibers are known to inhibit the growth of pathogens by preventing their adhesion to epithelial cells. Blocking pathogenic adhesion to epithelial cells may be beneficial because it prevents pathogen-induced epithelial damage^118^. Several studies have demonstrated that pectins and pectic-oligosaccharides (POS) have the ability to block the adhesion of pathogens to epithelial cells^99,119–121^. POS are known to reduce the adhesion of pathogenic bacteria, such as *Escherichia coli, Salmonella Typhimurium, Listeria monocytogenes*, and *Campylobacter jejuni*, to epithelial cells^122–124^. This anti-adhesive effect of pectins and POS on pathogens was higher with pectins with a lower molecular weight or DM3 or DM5 pectins^119,121^.

In contrast to the anti-adhesive effects of pectins on pathogens, stimulating adhesion of commensal bacteria to epithelial cells may be favorable, as commensal bacteria may compete with pathogens for attachment or secrete other beneficial products^118^. Several studies have shown that pectins or pectic oligosaccharides enhance the adherence of commensal or probiotic Lactobacilli strains to epithelial cells^122,124^. The effects were species dependent, as pectins enhanced the adhesive effects of some *Lactobacillus* species, such as *Lactobacillus plantarum* 0981, 0995 or *Lactobacillus brevis* 0983, whereas pectins decreased the adhesion of other *Lactobacillus* species, such as *Lactobacillus paracasei* 0985, *Lactobacillus* plantarum 0989, 0990, and 0996^122^. The structural characteristics were, however, not specified in those studies. The exact mechanisms by which pectins or dietary fibers affect bacterial attachment are not fully understood and are thought to be multifaceted^125^. Fibers may resemble the saccharides from glycoproteins on epithelial cells that pathogens normally adhere to^126^. Furthermore, pectins may target other mechanisms in epithelial cells, including the regulation of transcription factors, chaperone proteins, glycosidic hydrolysates or other adhesion-related proteins^125^.

## Concluding remarks and future perspectives

**Fig. 4:**
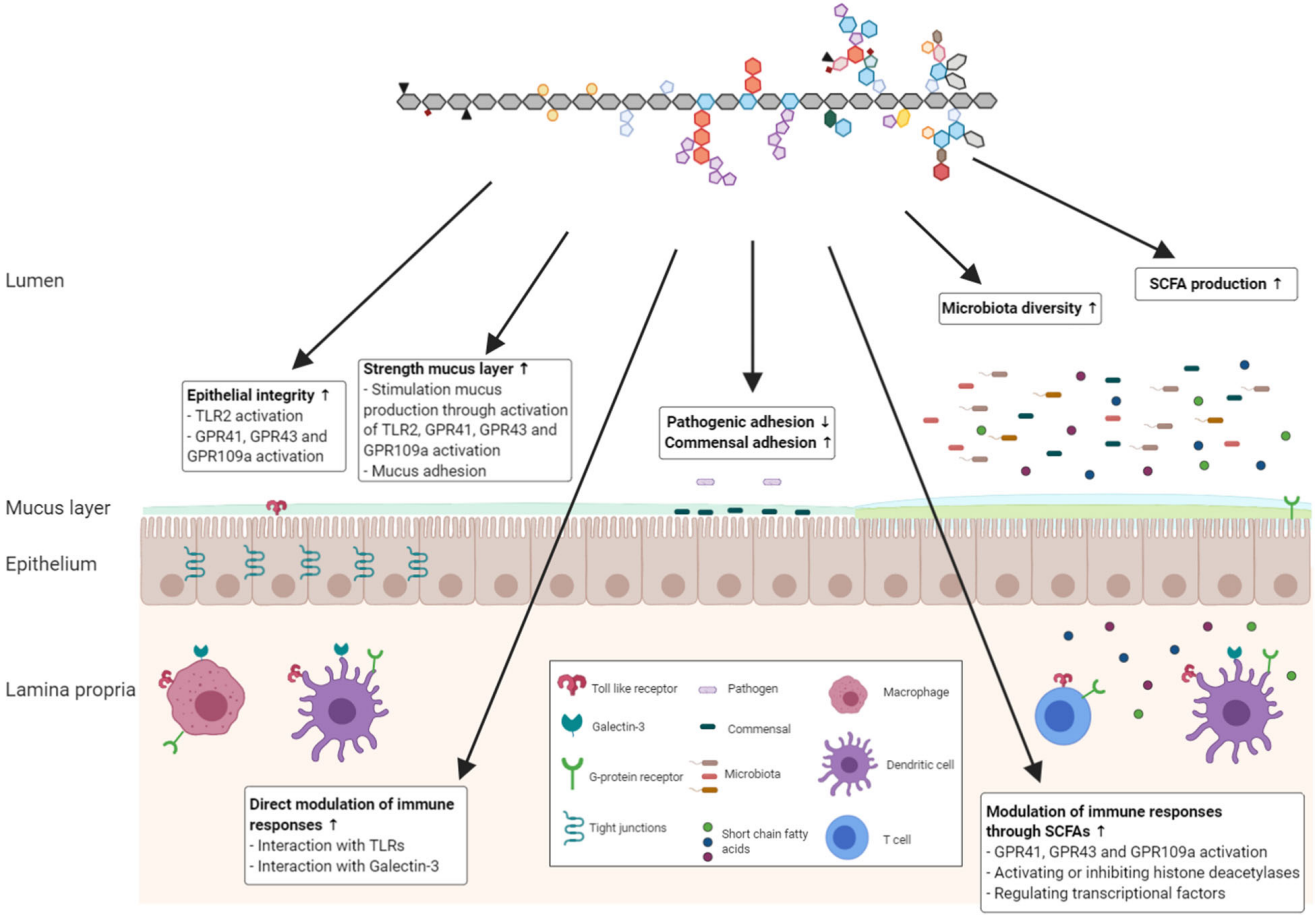
Influence of pectins on the gastrointestinal immune barrier. Pectins can influence the gastrointestinal immune barrier in a microbiota-dependent (indirect effects) and microbiota-independent (direct effects) manner. Direct effects include strengthening of the mucus layer, stimulation of epithelial integrity or modulation of immune responses. Pectins can exert direct effects through interaction with galectins or TLRs. Indirect effects include stimulation of microbial diversity, production of SCFAs, favoring adhesion of commensals to epithelial cells or anti-adhesive effects of pathogens to epithelial cells. SCFAs may stimulate epithelial integrity and mucus secretion by binding to GPR41, GPR43, or GPR109a. Immune responses are also influenced by SCFAs, which may interact with GPR41, GPR43, or GPR109a, activate or inhibit histone deacetylases or regulate transcription factors.

This review discusses the current knowledge on the impact of specific pectin structures on the intestinal immune barrier (Fig. 4). Several studies suggest that multiple structural characteristics of pectins exhibit diverse effects that enhance the intestinal immune barrier through a direct, microbiota-independent interaction with immune cells. Pectins may enhance the intestinal immune barrier by strengthening the mucus layer, enhancing epithelial integrity or activating and inhibiting macrophage and DC responses, which may be governed through interactions with PRRs^30,41,64–66,70–73,76,77^. However, most of these studies were performed in vitro. Some in vivo studies have confirmed the microbiota-independent effects of pectins on the gastrointestinal immune barrier. A recent study showed that pectins ameliorate TNSB-induced colitis in a microbiota-independent manner, as pectins had colitis-attenuating effects after deletion of microbiota by antibiotics^127^. Moreover, to show the direct microbiota-independent effects of pectins on the gastrointestinal immune barrier, it is recommended to perform in vivo studies with germ-free mice, such as those previously performed by Fransen et al., for inulin-type fructans^128^. Furthermore, additional experiments with human intestinal organoid models are needed to further extrapolate these direct effects of pectins on the gastrointestinal immune barrier of humans^129^.

In addition, the ability of pectins to reinforce the gastrointestinal immune barrier by impacting the gut microbiota and its fermentation products was reviewed. Specific pectin structures may contribute to intestinal homeostasis by stimulating the growth of microbial communities and enhancing their metabolic activity or by affecting the attachment of bacteria to epithelial cells^18,24,99,100^. However, due to the individual differences of the human microbiota, achieving similar effects of the pectin structures in humans may be challenging^130^. To better understand the microbiota-dependent impact of pectins on the gastrointestinal immune barrier, human studies are needed in which diet, age, gender, use of medication and host milieu are controlled^131^.

The dietary fiber pectin is a complex molecule with many structural characteristics^25^. Most studies that investigated the health effects of pectins used pectins with a combination of several structural characteristics. It is therefore hard if not impossible to determine which specific structural patterns of pectins induced health-promoting effects in these studies. To better understand how specific pectin structures can stimulate the intestinal immune barrier, future research should address the following: (1) The impact of the degree of single structural components of pectins (i.e., DM or acetylation, arabinose or galactose) on the gastrointestinal immune barrier; (2) The impact of the distribution of a single structural component (i.e., degree of blockiness) of pectins on the gastrointestinal immune barrier; (3) The combined impact of the degree and distribution of single structural characteristics of pectin on the gastrointestinal immune barrier. This may lead to the development of specific pectin formulations with beneficial effects on the gastrointestinal immune barrier in specific target groups. Because of its wide variety of structural features and specific health effects, pectin is a molecule that can be tailored for personalized medicine. Pectins may, for instance, be useful in preventing or restoring dysbiotic microbiota compositions associated with obesity, inflammatory bowel disease and metabolic syndrome^94^. Several studies have shown that supplementation of dietary fibers can prolong remission periods in patients with IBDs^132,133^, but no human studies have demonstrated that dietary fibers can be used to treat active IBDs^134^, suggesting that dietary fibers can prevent but not cure IBD symptoms. In Crohn’s disease, ulcerative colitis and IBS food processing are dysregulated and might lead to disturbed fermentation of dietary fibers with possible negative effects on disease progression as a consequence^134^. This should always be considered when administering dietary fibers in these disorders. Furthermore, the microbiota-independent effects of pectins, including reducing mucositis by blocking TLR2-1, may be useful in limiting chemically induced intestinal inflammation after chemotherapeutic or radiotherapeutic treatments^30,135^. Pectin-based nutraceutical or functional foods may therefore be used to limit the development of diseases and maintain health in a microbiota-dependent and microbiota-independent manner.
